# The Medical Society of the County of Erie, February 19th

**Published:** 1912-03

**Authors:** 


### SOCIETY PROCEEDINGS

### The Medical Society of the County of Erie, February 19th

Dr. Julius Y. Cohen and Dr. Clayton W. Greene were elected to membership.

Dr. George E. Stilson, formerly of 438 Potomac Avenue, Buffalo, but now residing at Niagara Falls, was granted a transfer to the Niagara County Society.

The amendment regarding contract practice was reported by Dr. Wisner R. Townsend, Secretary of the State Society, as disapproved in its present form. The entire question was referred to the Committee on Contract Work.

. Dr. William H. Thornton moved that a committee of, at least, five members be appointed by the President to take into consideration the establishment of a collection department for our society and that the President and Treasurer be two of its members. The President, later, appointed, as such committee, Dr. William H. Thornton, Chairman and Drs. F. W. Filsinger, E. L. Frost, H. K. DeGroat, Treasurer A. T. Lytle and President T. H. McKee.

A memorial on the death of the late Dr. James S. Smith, presented by Dr. W. T. Getman of the Committee on Necrology, will be found in the Obituary Department.

Scientific program:

"Hemorrhage and its Treatment," by Dr. F. C. Busch

"General Conditions in the Treatment of Surgical Tuberculosis with Tuberculin," by Dr. Norman K. McLeod.

"The Pictorial Accessory Cavities" (illustrated by Stereopticon), by Dr. George F. Cott.

